# Preparation of Chitosan Coated Magnetic Hydroxyapatite Nanoparticles and Application for Adsorption of Reactive Blue 19 and Ni^2+^ Ions

**DOI:** 10.1155/2014/273082

**Published:** 2014-02-02

**Authors:** Van Cuong Nguyen, Quoc Hue Pho

**Affiliations:** Department of Chemical Engineering, Industrial University of Ho Chi Minh City, 12 Nguyen Van Bao Street, Go Vap, Ho Chi Minh City 70000, Vietnam

## Abstract

An adsorbent called chitosan coated magnetic hydroxyapatite nanoparticles (CS-MHAP) was prepared with the purpose of improvement for the removal of Ni^2+^ ions and textile dye by coprecipitation. Structure and properties of CS-MHAP were characterized by scanning electron microscopy (SEM), X-ray diffraction (XRD), Fourier transform infrared spectroscopy (FTIR), and vibrating sample magnetometer (VSM). Weight percent of chitosan was investigated by thermal gravimetric analysis (TGA). The prepared CS-MHAP presents a significant improvement on the removal efficiency of Ni^2+^ ions and reactive blue 19 dye (RB19) in comparison with chitosan and magnetic hydroxyapatite nanoparticles. Moreover, the adsorption capacities were affected by several parameters such as contact time, initial concentration, adsorbent dosage, and initial pH. Interestingly, the prepared adsorbent could be easily recycled from an aqueous solution by an external magnet and reused for adsorption with high removal efficiency.

## 1. Introduction

Water that contains heavy metals and dye wastes represents one of the most problematic pollution groups because it can cause serious threat to human beings, animals, and plants. The wastewater was discharged from several industrial actives such as plating, metallurgy, and dyeing. Dyestuffs and pigments are popularly used in different fields in industries such as textile, paper, rubber, plastic, leather, cosmetics, food, and drug. The synthetic dyes and pigments are toxic, nonbiodegradable, and carcinogenic due to aromatic rings in the structures. Additionally, heavy metals such as Ni^2+^ and Cu^2+^ ions are known to be essential trace metals to human beings but high intake of Ni^2+^ and Cu^2+^ ions can also cause health problems like gastrointestinal disturbance, liver impaired failure, and kidney failure. However, it is not easy to treat the wastewater of dyes and heavy metals to become harmless to human and media surroundings [1, 2]. Recently, problems of removing dyes and heavy metals from wastewater in the industry have been becoming an important and significant field in wastewater treatment. There are many wastewater treatment methods used for dyes and heavy metals removal from wastewater [3, 4]: chemical treatment [5], biological method [6], physicochemical method [7], electrochemical process [8], adsorption [9], and photocatalytic degradation [10]. One of these methods called the adsorption method has become one of the most effective and feasible technique. In the adsorption technique, activated carbon has been reported to be the most effective material but is an expensive material and its use for applications of pollution control cannot be justified [11, 12]. Therefore, tendencies to use materials that are natural, biodegradable, cheap, renewable, and environmentally-friendly were supported to research by experts.

Recently, chitosan (CS) has been known as a novel biomaterial that scientists pay attention to. It has been described as a nontoxic, biodegradable, and biocompatible polymer with very interesting biological properties such as wound dressing, drug delivery systems, and tissue engineering [13–15]. However, chitosan has unexpected properties that makes itself become limited to use for adsorption such as poor mechanical strength, low specific gravity, easy agglomeration, or gel formation, and insufficient solubility in dilute acids largely limits its widespread applications for environmental pollutant removal [16, 17]. Therefore, chitosan is often used to combine with other materials to improve their unexpected properties. To pave the way for using CS for hazardous metals and dyes removals, several attempts have been made to improve the mechanical strength and adsorption efficiency by immobilization of CS on rigid inorganic materials. Hydroxyapatite [Ca_10_(PO_4_)_6_(OH)_2_, HAP], a very well-known material in bones and teeth, is combined with chitosan to create a novel composite that ameliorates properties of chitosan and is applied in biological and medical fields such as implant coating and bone tissue engineering [18, 19]. In addition, hydroxyapatite has a porous structure that makes it become widely used as an adsorbent for removal of heavy metals and dyes.

Applications of combining chitosan with hydroxyapatite as a novel, powerful material for adsorption have been reported in previous article [20, 21]. However, previous materials were not to be used popularly in industries due to its impossibility of renewal. Therefore, in this study the material was ameliorated by adding magnetic nanoparticles (Fe_3_O_4_) into the composite of chitosan and hydroxyapatites with the purpose of increasing their usage effectiveness through the magnetic separation. In this study, we propose to synthesize the magnetic composite of chitosan and hydroxyapatite and explore the possibility of using prepared magnetic composite as an adsorbent for the removal of organic textile dyes and heavy metals from aqueous solutions.

## 2. Materials and Methods

### 2.1. Materials

Chitosan (90% deacetylation) was prepared in our lab. Reactive blue 19 (RB19) was donated by Thanh Cong Textile Company. All chemicals were analytical grade and used without further purification and all solutions were prepared with deionized water.

### 2.2. Preparation of Magnetic Hydroxyapatite Nanoparticles (MHAP)

In this study, the MHAP material was synthesized by coprecipitation method according to the previous report with some modification [22]. First, FeCl_2_·4H_2_O and FeCl_3_·6H_2_O salts were used in the molar ratio 2 : 1. In brief, determined amounts of FeCl_2_·4H_2_O (1.85 mmol) and FeCl_3_·6H_2_O (3.7 mmol) were dissolved in 30 mL of deoxygenated water under a nitrogen atmosphere at room temperature then 10 mL of 25% NH_4_OH solution were added to the resulting solution under a magnetic stirrer (3000 rpm) for 15 min. Then, Ca(NO_3_)_2_·4H_2_O (33.7 mmol) and (NH_4_)_2_HPO_4_·4H_2_O (20 mmol) dissolved in 50 mL water and were added to the previously mentioned solution, then the pH of the resulting solution was adjusted to 11. The precipitation was formed simultaneously and stirred for 30 min at room temperature and increased to 90°C for 2 hr. The mixture was cooled to room temperature and aged for 12–24 h without stirring. The obtained precipitate was separated by a magnet, washed repeatedly with deionized water until neutrality, then dried in a drying oven at 90°C, and grinded with mortar.

### 2.3. Preparation of Chitosan Coated Magnetic Hydroxyapatite Nanoparticles (CS-MHAP)

In order to coat chitosan onto surface of MHAP, chitosan reacted with glutaraldehyde to cross-link bonds [23, 24]. An appropriate amount of chitosan (1.25 g) was dissolved in 125 mL acetic acid (1%) in a 500 mL beaker containing an amount of the as-prepared MHAP. The mixed solution was stirred continuously for 1 h in order to disperse regularly MHAP in chitosan solution. Then, 0.5 mL glutaraldehyde 25% was added into the mixture to form gel. After completion of the reaction, the pH in the system was adjusted to 9 using 2 M NaOH solution. The mixture was stirred and heated on a water bath for 3 h at 60°C. The magnetic beads were formed and washed with acetone and distilled water. Finally, the CS-MHAP materials were dried at 80°C.

### 2.4. Characterization

An X-ray diffraction was carried out in X'Pert Pro MRD (PANanalytical, The Netherlands) at room temperature with Cu-K*α* radiation (*λ* = 0.154 nm) and an incident angle (2*θ*), ramping from 2 to 80° at a rate of 50 seconds/step. Magnetic measurement of the product was conducted on a vibrating sample magnetometer (VSM-5 Model, MicroSense Co., Ltd.). Scanning Electron Microscope (SEM) and Fourier transform infrared spectroscopy (FTIR) were used to characterize the morphology and structure of prepared material.

### 2.5. Adsorption of Reactive Blue 19 Experiments

0.1 g CS-MHAP composite was added into 50 mL RB19 solution with different initial concentrations (50, 75, 100, and 150 mg L^−1^). A stirrer was used to mix the solutions. The samples from suspension were separated by a permanent magnet. Concentration of samples was determined using a UV-Vis spectrophotometer at *λ* = 592 nm.

#### 2.5.1. Effect of Contact Time and Initial Concentration

The effect of equilibration of adsorbents was studied in different time intervals ranging from 10 to 240 min with initial RB19 concentrations of 50, 75, 100, and 150 mg·L^−1^. The aqueous samples were taken at determined intervals and the concentrations of dye were measured at 592 nm, using UV-Vis spectrophotometer. Adsorptive efficiency of CS-MHAP and equilibrium adsorption capacity *q*_*e*_ (mg·g^−1^) were calculated as follows [25]:

(1) $$\begin{array}{c} R=\frac{\left(C_{0}-C_{e}\right)}{C_{0}}\times 100\%,\\ q_{e}=\frac{\left(C_{0}-C_{e}\right)\times V}{m}, \end{array}$$

where *C*_0_ and *C*_*e*_ are the initial and equilibrium concentration (mg·L^−1^), respectively, *m* is dosage of CS-MHAP (mg), and *V* is volume of solution (L).

#### 2.5.2. Effect of CS-MHAP Dosage

Different amounts of CS-MHAP (0.025 to 0.1 g; 0.5 to 2 g L^−1^) and 50 mL of 50 mg L^−1^ RB19 solutions were placed in a 100 mL beaker and stirred regularly in different time intervals. After separation, the concentrations of dye in the solutions were determined.

#### 2.5.3. Effect of pH Solution

For the same initial concentration, equilibration time and adsorbent amount were investigated as a function of pH. The initial pH values were adjusted 4, 6, 8, 10, and 12 using 0.1 M HCl or 0.1 M NaOH solution. After equilibration time, the suspensions were separated and the residual RB19 concentrations were measured.

### 2.6. Adsorption of Ni^2+^ Ions Experiments

Stock solutions (1000 mg L^−1^) of Ni^2+^ were prepared for adsorption experiments. The solutions of different concentrations used in various experiments were obtained by dilution of the stock solutions. 0.5 g CS-MHAP was added into the 50 mL Ni^2+^ solution with various initial concentrations. The reaction solution was mixed by a stirrer. The samples from the suspension were collected at regular intervals of time, separated by a permanent magnet. Concentrations of samples of Ni^2+^ ion were determined by using standard methods recommended for examination of water and wastewater [26]. Adsorption efficiency of CS-MHAP and equilibrium adsorption capacity *q*_*e*_ (mg g^−1^) were calculated as follows (1). The amount of adsorption at time *t*, *q*_*t*_ (mg/g), is calculated by *q*_*t*_ = (*C*_0_ − *C*_*t*_)∗*V*/*W* where *C*_0_ and *C*_*t*_ (mg/L) are the liquid-phase concentrations of Ni^2+^ ions at initial and any time *t*, respectively; *V* is the volume of the solution (L); *W* is the mass of dry adsorbent used (g).

#### 2.6.1. Effect of Contact Time and Initial Concentration

The effect of contact time on each metal sorption was studied in different time intervals ranging from 30 min to 6 h with 25, 50, 75, and 100 mg L^−1^ initial metal concentrations. After the completion of the reaction, the CS-MHAP adsorbents were separated by a permanent magnet. Residual concentrations of Ni^2+^ ions were determined.

#### 2.6.2. Effect of CS-MHAP Dosage

Different amounts of MNHAP-CS (0.1 to 0.5 g; 2 to 10 g L^−1^) and 50 mL of 50 mg L^−1^ metal solutions were placed in 100 mL beakers and stirred regularly in different time intervals. After separation, the final concentrations of Ni^2+^ in the solutions were measured.

#### 2.6.3. Effect of pH Solution

The effect of pH on the adsorption of Ni^2+^ ions was examined by mixing 0.5 g composite with 50 mL of Ni^2+^ ions solution (50 mg L^−1^), equilibrium time 240 min, and the pH ranging from 4 to 7. The initial pH values were adjusted from 4 to 8, using 0.1 M HCl or 0.1 M NaOH solution. After equilibration time, the suspensions were separated and the residual metal concentrations were analyzed.

## 3. Results and Discussion

### 3.1. Properties of Prepared CS-MHAP Adsorbent

**Figure 1:**
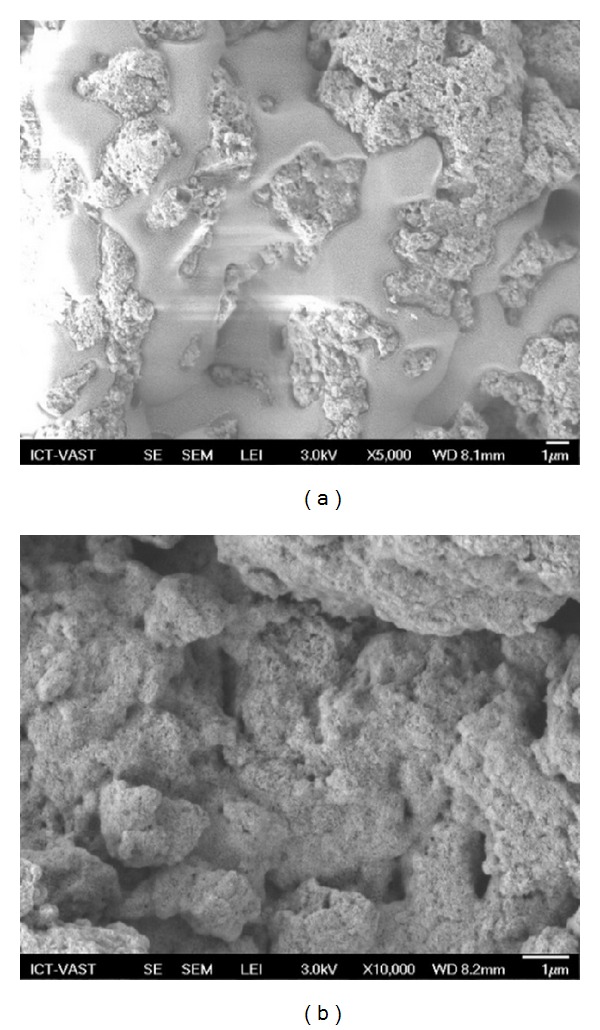
SEM images of prepared CS-MHAP (a) 5000× and (b) 10000×.

The scanning electron microscope (SEM) micrograph of CS-MHAP is shown in Figure 1. The result of SEM micrograph shows that composite surface is rough and has porous structure with holes and small openings on the surface, resulting in this prepared material which has a good adsorption capacity. The homogeneously distributed pore structure of MHAP nanoparticles was indicated in Figure 1(a) also supported by the high porosity and high open pore content. The microstructure obtained by SEM (Figure 1(b)) for CS-MHAP composite prepared by gel-formation showed that the MHAP nanoparticles are relatively dispersed in the chitosan matrix. As shown in Figure 1, the MHAP material has two different solid phases meaning that there is a phase of magnetic and another phase of hydroxyapatite.

**Figure 2:**
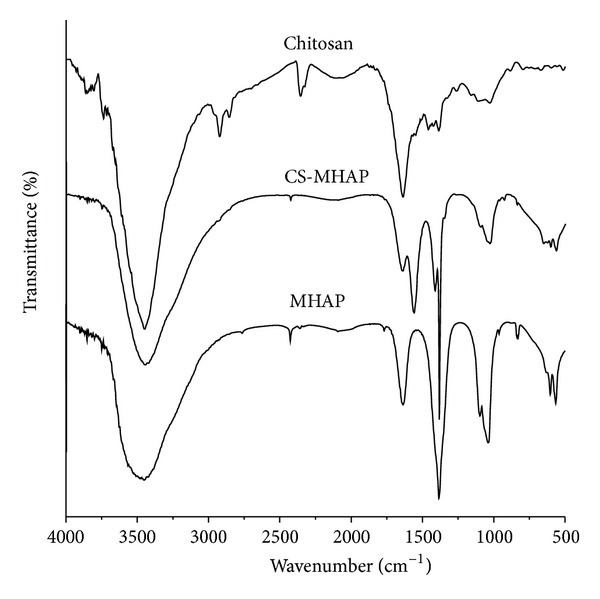
FTIR spectrum of prepared materials chitosan, CS-MHAP, and MHAP.

The FTIR graphs of CS-MHAP and MHAP are shown in Figure 2. As can be seen, the adsorption bands at around 618 cm^−1^ correspond to the stretching and vibration of the lattice OH^−^ ions, while the bands of adsorbed water are shown at 3445.79 cm^−1^ and 1635.92 cm^−1^. The characteristic bands for PO_4_^3−^ represented at 560 cm^−1^ and 1096.64 cm^−1^. Moreover, several typical bands were assigned for existence of chitosan in structure of the CS-MHAP. The characteristic absorption bands appeared at around 3445 cm^−1^ corresponding to the stretching vibration of O–H and N–H bonds. The peaks at 2923 cm^−1^, 1641 cm^−1^, 1388 cm^−1^, and 1029 cm^−1^ are ascribed to C–H of alkyl group, C=O of amide I, –NHCO of amide III, and C–OH bond, respectively. The absorption peak of chitosan at 1560 cm^−1^ was assigned to the characteristic peak of NH_2_, while this peak was shifted to 1597 cm^−1^ in the composite. The appearance of new peak at 1597 cm^−1^ in FT-IR was related to the vibrations of protonated amine groups [27].

**Figure 3:**
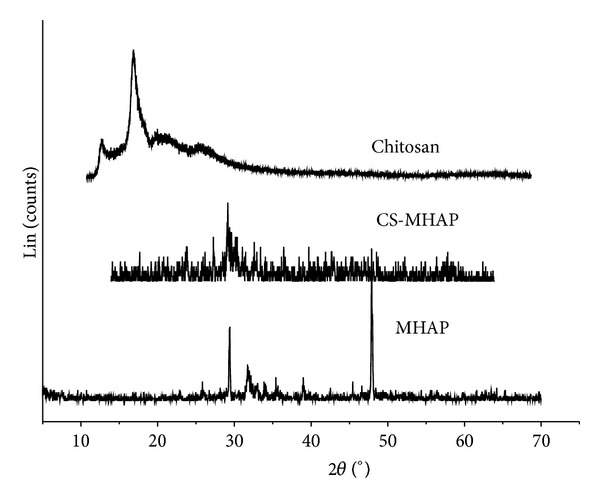
X-ray diffraction (XRD) pattern of prepared materials.

The XRD patterns of chitosan, MHAP, and CS-MHAP were shown in Figure 3. The straight base line and the not sharp peaks of the diffractogram confirmed that the CS-MHAP was not well crystallized as the MHAP due to the representation of chitosan coated on the surface of MHAP. Figure 3 also represents the peaks at 2**θ**  of 25.8°, 31.8°, 32.1°, 32.9°, 34°, 39.9°, 46.7°, and 49.4° for the existence of HA in the CS-MHAP. The peaks at 2**θ**  of 10.02°, 19.8°, and 21.9° indicate that chitosan is coated on MHAP. Furthermore, the peaks at 2**θ**  of 30°, 35.5°, and 63° are typical peaks for representation of Fe_3_O_4_ in the CS-MHAP. A similar assignment was reported previously [27].

**Figure 4:**
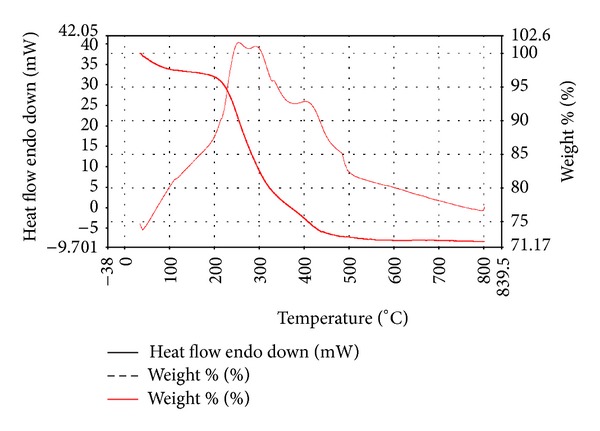
The thermogravimetric analysis (TGA) of CS-MHAP.

The thermogravimetric analysis (TGA) (Figure 4) was carried out in air at a heating rate of 10°C/min [28]. As can be seen, there are two characterized curves for the thermogravimetric analysis. These curves are the TG and DSC curves. The TG curve of CS-MHAP shows that the weight loss over temperature from 35 to 800°C was about 28% in two stages. The first weight loss was 3.052% indicating that mass of water or volatile compounds entrapped in prepared composite with temperatures from 35 to 175°C. Additionally, an endothermic peak could be seen from the DSC curve. The weight loss in the second stage from 175 to 800°C was 25%. This was mainly due to the decomposition of chitosan and an obvious peak was observed in DSC curve. Therefore, the residual compounds of prepared composite after heating at 800°C are Fe_3_O_4_ and hydroxyapatite nanoparticles (72% weight of CS-MHAP) due to their high melting points (over 1500°C).

**Figure 5:**
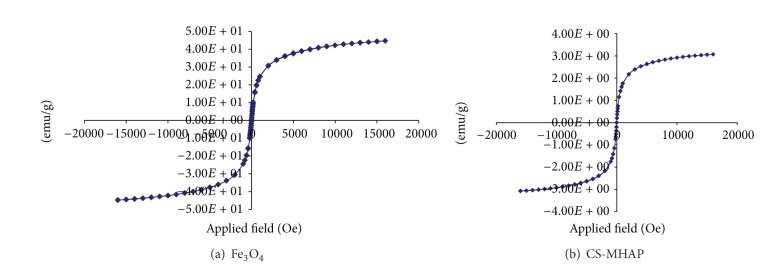
Magnetization curves of (a) Fe_3_O_4_ and (b) CS-MHAP.

Hysteresis loops of Fe_3_O_4_ and CS-MHAP were shown in Figure 5. The magnetite (Fe_3_O_4_) had saturated magnetization about 44.8 emu/g. However, the saturated magnetization of CS-MHAP was decreased to 3.1 emu/g. The results showed that saturated magnetization of Fe_3_O_4_ is much higher than that of CS-MHAP due to the influence of chitosan content and dispersion of Fe_3_O_4_ in a large number of hydroxyapatite nanoparticles in the composite MHAP. However, magnetic composite adsorbents could be quickly collected by applying an external magnet after adsorption. Additionally, the result indicates that saturated magnetization CS-MHAP could be expediently adjusted by changing additive dosage of chitosan, hydroxyapatite, and Fe_3_O_4_ nanoparticles.

### 3.2. Adsorption of RB19 Dye

#### 3.2.1. Effect of Contact Time and Initial Concentration

**Figure 6:**
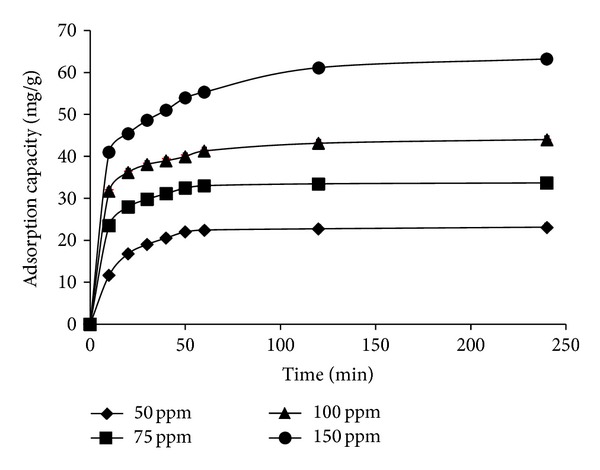
Adsorption kinetics of RB19 onto CS-MHAP at different initial concentrations (0.1 g CS-MHAP; solution volume: 50 mL and pH = 6).

**Figure 7:**
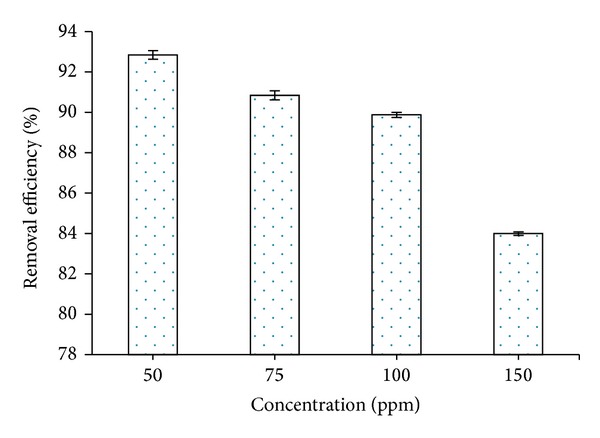
Adsorption efficiency of CS-MHAP at different concentrations of RB19 (0.1 g CS-MHAP; solution volume: 50 mL and pH = 6).

Figure 6 showed the effect of contact time and initial concentrations on the adsorption capacities of RB19 onto CS-MHAP. As can be seen, when the time increased from 10 to 240 min, the adsorption capacity of RB19 was directly proportional to the contact time. Moreover, the adsorptive process reached equilibrium at 240 min for concentration of 150 ppm and decreased with decreasing initial concentrations of dye (60 min for concentration of 50 and 75 ppm and 120 min for 100 ppm). Additionally, an increase in the initial dye concentration leads to an increase in the adsorption capacity of the dye onto CS-MHAP. The adsorption efficiency decreased gradually with an increase of dye concentration. Particularly, adsorption efficiency was 93% at concentration of 50 ppm and decreased to 91.2, 90.5, and 84% for concentrations of 75, 100, and 150 ppm, respectively (Figure 7). These results confirmed that initial dye concentrations played an important role in the adsorption of organic dyes onto the CS-MHAP. The adsorption efficiency increased with increase in the initial dye concentration. This may explain due to the increase in the driving force of the concentration gradient.

#### 3.2.2. Effect of pH Solution

**Figure 8:**
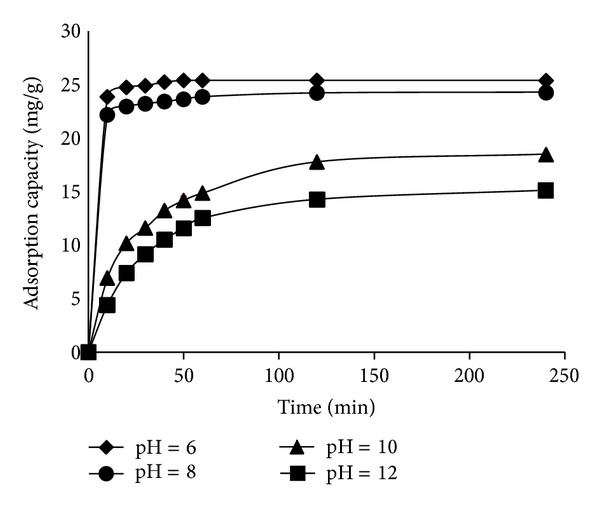
Effect of initial pH on the adsorption of RB19 onto CS-MHAP (0.1 g CS-MHAP; solution volume: 50 mL and concentration of 50 ppm).

The effect of initial pH on adsorption capacity of RB19 onto CS-MHAP was studied at different pH ranging from 6 to 12. The results were represented in Figure 8. These results expressed that the adsorption capacity of RB19 increased with the decreasing of pH. As seen, it is clear that the adsorption capacity at acidic solution (at pH = 6; *q*_*o*_ = 25.386 mg/g) is higher than those of basic solutions (at pH = 8, 10, and 12; *q*_*o*_ = 24.3, 18.5, and 15.1 mg/g, resp.). This is due to the fact that at a lower pH the –NH_2_ groups of chitosan were protonated by H_3_O^+^ ions in acidic solution which yields positively charged –NH_3_^+^ groups. Moreover, the RB19 is an anionic dye, which can be adsorbed on positively charged surface of CS-MHAP. On the contrary, when the pH of the solution increases, high OH ions accumulate on the adsorbent surface. Therefore, electrostatic interaction between negatively charged adsorbent surface and anionic dye molecule decreases the adsorption of dye molecular on the surface of CS-MHAP. Moreover, the adsorption capacity at pH 4 was lower than that of pH 6 due to the solubility of chitosan shells in acidic media [17].

#### 3.2.3. Effect of CS-MHAP Dosage

**Figure 9:**
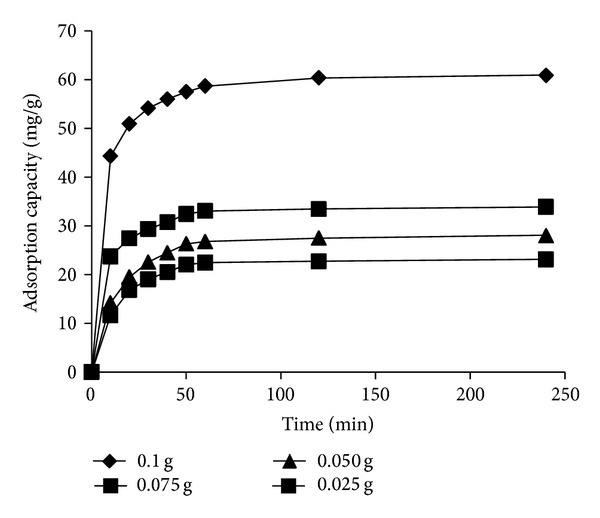
Adsorption kinetics of RB19 onto CS-MHAP at different amounts of adsorbent (solution volume: 50 mL; pH = 6 and concentration of 50 ppm).

Adsorption dosage is an important parameter because it determines the capacity of an adsorbent for a given initial concentration of adsorbate. As shown in Figure 9, the amount of RB19 adsorbed increased rapidly with the increasing of CS-MHAP amount at first 50 min and then it increased slowly from 50 to 120 min. The adsorption capacity reached a maximum equilibrium at 240 min. Additionally, it was found that the adsorption capacity rises with an increase in the weight of adsorbents; this is because the adsorption capacity depends on the external surface of the adsorbent increases with a large mass. The adsorption capacity of RB19 dye onto CS-MHAP is 60.9 mg/g for 0.1 g of CS-MHAP and decreased to 23.1 mg/g for 0.025 g of CS-MHAP (Figure 9).

**Figure 10:**
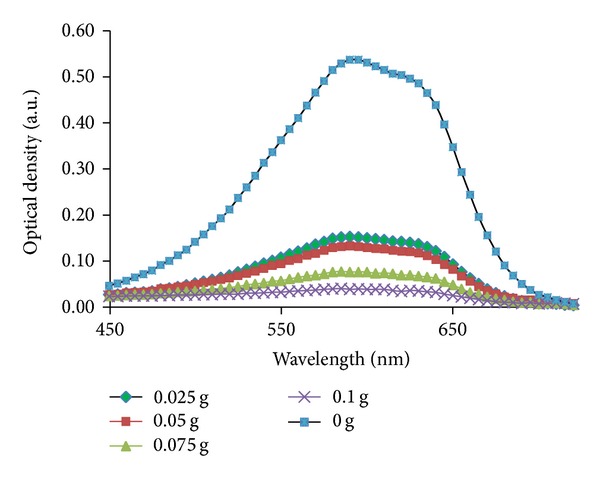
The change in absorption spectra of RB19 using various amounts of CS-MHAP (solution volume: 50 mL; pH = 6 and concentration of 50 ppm).

As shown in Figure 10, the adsorption of RB19 at 592 nm decreased with increasing the MHAP amount. The disappearance of adsorption at 592 nm was observed at dosage of 0.1 g; this confirmed that most of RB19 was adsorbed under experiment condition.

### 3.3. Adsorption of Ni^2+^ Ions

#### 3.3.1. Effect of Contact Time and Initial Concentration

**Figure 11:**
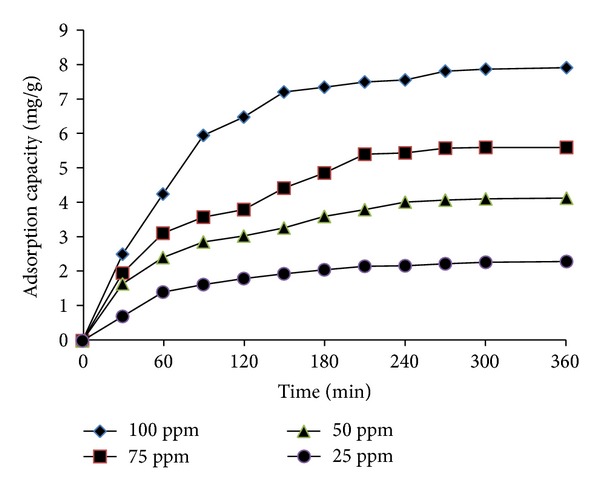
Effect of contact time on adsorption of Ni^2+^ ions onto CS-MHAP (0.5 g CS-MHAP; solution volume: 50 mL and pH = 6).

**Figure 12:**
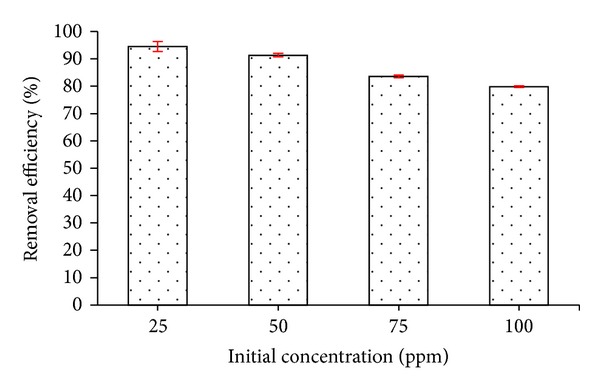
Removal efficiency of different initial Ni^2+^ ion concentrations onto CS-MHAP (0.5 g CS-MHAP; solution volume: 50 mL and pH = 6).

The effect of contact time and initial concentration of Ni^2+^ ions was presented in Figure 11. As can be seen, adsorption capacity of Ni^2+^ was positively correlated with increasing Ni^2+^ ions concentrations from 25 to 100 ppm and contact time before equilibrium was attained. Additionally, the results indicated that equilibrium time increased with increasing initial concentration of metal ions. At the concentration of 25 ppm the adsorption phenomena occurred quickly and almost reached equilibrium after 180 min and increased to 300 and 360 min for concentrations of 50 and 100 ppm, respectively. As observed, the removal efficiency was 96.5% at a concentration of 25 ppm; however, the removal efficiency decreased to 83.5% at a concentration of 100 ppm (Figure 12). The adsorption mechanism of Ni^2+^ was explained by a combination of 2 processes: (1) adsorption of Ni^2+^ ions onto surface of chitosan by a complex reaction due to –NH_2_ and –OH groups of chitosan [29] and (2) adsorption of Ni^2+^ ions onto the surface of hydroxyapatite nanoparticles by exchanging with Ca^2+^ ions [30].

#### 3.3.2. Effect of pH

**Figure 13:**
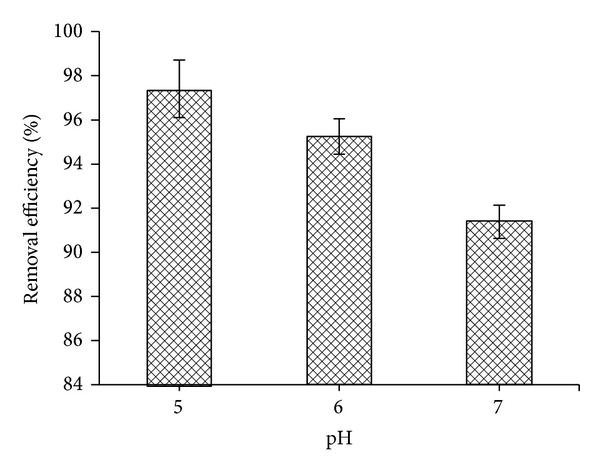
The effect of pH on adsorption capacity of Ni^2+^ ions onto CS-MHAP (0.5 g CS-MHAP; solution volume: 50 mL and concentration of 50 ppm).

As known, the pH affects the availability of metal ions in solutions and the metal binding sites of the adsorbent. The effect of pH on the removal efficiency of Ni^2+^ ions onto CS-MHAP was determined at the range of pH from 4 to 7. The removal efficiency of Ni^2+^ ions onto CS-MHAP decreased when the pH value increased from 5 to 7. It should be noted that when the pH value is higher than 7, the adsorption amount decreased dramatically, which was attributed to the fact that Ni^2+^ ion started to precipitate leading to the reduction of Ni^2+^ in the solution. The removal efficiencies were 97.4, 95.6, and 91.1% for pH of 5, 6, and 7, respectively (Figure 13).

#### 3.3.3. Effect of CS-MHAP Amount

**Figure 14:**
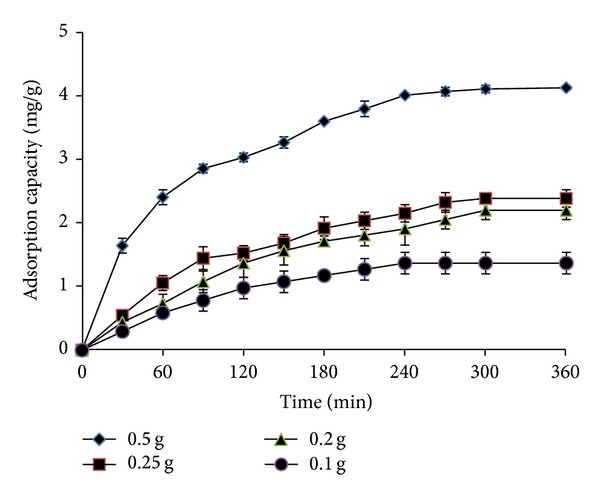
The relationship between adsorption capacity of Ni^2+^ ions and CS-MHAP dosage (solution volume: 50 mL; pH = 5 and concentration of 50 ppm).

In this present study, the dependence of the adsorption capacity of Ni^2+^ ions onto CS-MHAP amount was investigated by varying dosage of adsorbent from 0.1 to 0.5 g, while keeping other parameters constant (Figure 14). As the adsorption phenomena of RB19, the adsorption capacity of Ni^2+^ ions onto CS-MHAP augmented rapidly and the time to reach equilibrium was more quick when dosage of adsorbents increased. Particularly, at equilibrium time, the adsorption capacity of Ni^2+^ ions was 4.3 mg/g with 0.5 g dosage of the adsorbent and decreased to 1.4 mg/g for 0.1 g CS-MHAP. Additionally, the removal efficiencies were increased from 19.8 to 91.2% when the dose of adsorbents was increased from 0.1 to 0.5 g. This could be explained by the fact that the higher dose of adsorbents in the solution, the greater availability of exchangeable sites for the ions.

### 3.4. Comparative Adsorption

**Figure 15:**
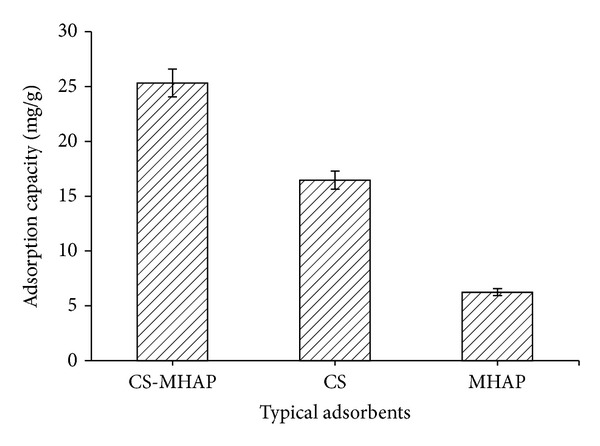
The adsorption of typical adsorbents for adsorption of RB19 (solution volume: 50 mL; pH = 6 and concentration of 50 ppm).

**Figure 16:**
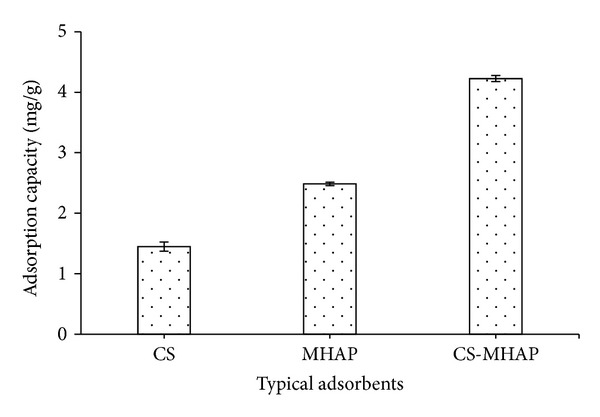
Adsorption comparison of typical adsorbents for adsorption of Ni^2+^ ions (solution volume: 50 mL; pH = 5 and concentration of 50 ppm).

The variances of adsorption capacity of RB 19 and Ni^2+^ ions for different adsorbents were shown in Figures 15 and 16. As a result, CS-MHAP represented the most effectiveness for adsorption of metal ions and dye among the various adsorbents (chitosan and magnetic hydroxyapatite nanoparticles). The results indicated that combination of CS and HA into composite leads to adsorption capacity of CS-MHAP which remarkably improved. The adsorption amounts for MHAP, chitosan, and CS-MHAP were 6.2, 16.4, and 26.4 mg/g, respectively. The adsorption capacity of Ni^2+^ ions CS-MHAP was 4.3 mg/g and decreased to 2.5 and 1.4 mg/g for MHAP and chitosan, respectively. In addition, the magnetic composite adsorbents also showed the magnetic property and significant improvement of the separability from aqueous solutions after adsorption.

## 4. Conclusions

In this present study, a novel magnetic composite of chitosan and hydroxyapatite nanoparticles was successfully prepared by simple precipitation method. Then, the prepared composite was characterized by XRD, FT-IR, TGA, SEM, and VSM. The adsorption of heavy metals and dyes onto the prepared magnetic composite was investigated in a batch experiment. The various parameters such as contact time, initial concentrations, pH, and adsorbent dosage showed significantly effects on the adsorption capacity of Ni^2+^ ions and dye. Maximum removals of RB19 and Ni^2+^ ions onto CS-MHAP were at pH 5.0 and pH 6, respectively.
